# Self-Supervised Learning and Multi-Sensor Fusion for Alpine Wetland Vegetation Mapping: Bayinbuluke, China

**DOI:** 10.3390/plants14203153

**Published:** 2025-10-13

**Authors:** Muhammad Murtaza Zaka, Alim Samat, Jilili Abuduwaili, Enzhao Zhu, Arslan Akhtar, Wenbo Li

**Affiliations:** 1 State Key Laboratory of Ecological Safety and Sustainable Development in Arid Lands, Xinjiang Institute of Ecology and Geography, Chinese Academy of Sciences, Urumqi 830011, China; 2 University of Chinese Academy of Sciences, Beijing 100049, China; 3 China-Kazakhstan Joint Laboratory for RS Technology and Application, Al-Farabi Kazakh National University, Almaty 050012, Kazakhstan; 4 CAS Research Center for Ecology and Environment of Central Asia, Urumqi 830011, China; 5 National Engineering Technology Research Center for Desert-Oasis Ecological Construction, Urumqi 830011, China; 6 Department of Electrical, Computer and Biomedical Engineering, University of Pavia, 27100 Pavia, Italy

**Keywords:** remote sensing of wetlands, self-supervised learning, multi-modal data fusion, vegetation mapping, alpine wetlands (Bayinbuluke, China), invasive plant species (IPS)

## Abstract

Accurate mapping of wetland vegetation is essential for ecological monitoring and conservation, yet it remains challenging due to the spatial heterogeneity of wetlands, the scarcity of ground-truth data, and the spread of invasive species. Invasive plants alter native vegetation patterns, making their early detection critical for preserving ecosystem integrity. This study proposes a novel framework that integrates self-supervised learning (SSL), supervised segmentation, and multi-sensor data fusion to enhance vegetation classification in the Bayinbuluke Alpine Wetland, China. High-resolution satellite imagery from PlanetScope-3 and Jilin-1 was fused, and SSL methods—including BYOL, DINO, and MoCo v3—were employed to learn transferable feature representations without extensive labeled data. The results show that SSL methods exhibit consistent variations in classification performance, while multi-sensor fusion significantly improves the detection of rare and fragmented vegetation patches and enables the early identification of invasive species. Overall, the proposed SSL–fusion strategy reduces reliance on labor-intensive field data collection and provides a scalable, high-precision solution for wetland monitoring and invasive species management.

## 1. Introduction

Wetland ecosystems play a crucial role in maintaining ecological balance and biodiversity [1]. Therefore, accurate mapping of these landscapes is vital for conservation and sustainable development [2]. Remote sensing has been widely used for wetland vegetation monitoring and hyperspectral imagery (HSI) offers distinct advantages through its fine spectral resolution which enables broad vegetation discrimination and detection of stable ecological changes [3]. However, the effectiveness of HIS is often limited by insufficient ground-truth data, particularly in heterogeneous wetlands with different land cover types [4]. Additional challenges include high spatial heterogeneity, temporal variability, fluctuating water levels, and spectrally similar plant species [5].

Another major challenge in wetland mapping arises from invasive plant species (IPS), which displace native vegetation, alter hydrological processes, and reduce biodiversity [6,7]. Moreover, from a remote sensing perspective, invasive plants are difficult to identify because their spectral similarity to co-occurring vegetation can obscure class separability. Patchy distribution and rapid spread also introduce temporal variability that complicates ecological monitoring [8,9]. Early and accurate identification of IPS is essential for the precision of wetland vegetation maps. This helps direct focused conservation and restoration efforts [10,11].

Although hyperspectral imagery (HSI) enhances vegetation discrimination by capturing hundreds of narrow bands [12] and spectral–spatial models to achieve better classification accuracy [13], traditional supervised learning (SL) approaches such as Support Vector Machines (SVM) and Random Forests (RF) have been used to classify wetlands [14]. In wetlands [15], these challenges highlight the limitations of purely supervised approaches and motivate the development of alternative strategies [16].

A viable approach is self-supervised learning (SSL), which mitigates data scarcity by deriving features from unlabeled data [17] and utilizing pretext activities to extract features pertinent for further classification [18]. Recent advancements in contrastive and predictive self-supervised learning, including global–local alignment and multiscale context modeling, have demonstrated robust efficacy in highly changeable land-cover environments. Methods such as BYOL [19], DINOv2 [20], and MoCo v3 [21] have exhibited robust performance in remote sensing applications characterized by a scarcity of labeled data [22]. Applying SSL to hyperspectral wetland imagery enables the extraction of high-level feature representations that capture both spatial and spectral vegetation patterns, along with other environmental features [23]. These representations are generated directly from the data, eliminating the need for predefined labels—particularly advantageous in wetland mapping, where annotated datasets are often unavailable [24]. These SSL-based features can then be integrated into supervised learning pipelines, such as DeepLabV3, a widely used semantic segmentation framework in remote sensing [25]. DeepLabV3 employs an atrous convolution architecture that facilitates the acquisition of multiscale contextual and spatial details, which is particularly advantageous due to the limited availability of annotated wetland datasets [26].

Additionally, SSL, SL, and data fusion (DF) are an essential process for improving classification accuracy. Such complementary spectral, spatial, and temporal resolutions can be separately accessed in different remote sensing datasets, allowing a more comprehensive representation of wetland ecosystems [27]. High-resolution imagery captures fine structural details [28], whereas high-temporal-resolution data elucidate seasonal and interannual changes [29]. Multispectral data add unique spectral information that improves the separability of vegetation types that may otherwise appear similar in a single dataset [30]. By integrating these heterogeneous sources, data fusion (DF) produces more robust and precise models for wetland vegetation classification [31]. For example, fusion hyperspectral dataset can enhance both spectral fidelity and spatial detail [32], particularly when supported by transformer-based spatial–spectral networks [33].

However, data-fused imaging frequently suffers from noise, particularly in wetlands, where mixed pixels and atmospheric influences compromise signal integrity. This work employs EN2N, which is a self-supervised Noise2Noise technique tailored for hyperspectral imaging. Analogous to SPEND, which eliminates spatially correlated noise in the absence of clear references [34], EN2N directly denoises noisy observations, maintaining spectral–spatial detail, and markedly enhancing the robustness and precision of wetland vegetation categorization.

This study demonstrated a framework that combines self-supervised learning (SSL) to extract supervised learning (SL) features to classify and utilize data fusion (DF) techniques to create better maps of wetland vegetation. There are some advantages to such a combination: with the use of SSL, it is possible to have fewer dependencies on manually annotated data and SL has a good classification capability, while DF improves the performance of the model due to synthesized complementary information by multiple sensors. Together, these methods offer a more reliable and accurate approach to monitoring and protecting wetlands. In addition, the improvement in methodology improves the framework for long-term monitoring, sustainable wetland management, and biodiversity conservation. These tools are badly required in light of the increasing human pressures from climate, land use, and human development. Furthermore, the research methodology also provides a viable solution to current problems and offers opportunities for future studies in remote sensing and ecological surveillance.

## 2. Results

### 2.1. Implementation Details

We employ a ResNet-50 as the online encoder for self-supervised pretraining. The encoder begins with a 7 × 7 convolution (stride =2 × 2, padding =3 × 3) whose input channels are adapted to the dataset’s full spectral depth ∁. Given an input patch $X\;\epsilon \;{\mathbb{R}}^{∁x64x64}$, the stem applies weights $W_{0}\in \;{\mathbb{R}}^{64x∁x7x7}$, after which features traverse the ResNet bottleneck blocks and an adaptive average pooling layer to yield a 2048-D representation, as shown in Equation (1).

(1) $$z=f_{0}\left(x\right)\epsilon \;{\mathbb{R}}^{2048}$$

A projection head $g_{\emptyset }\left(.\right)$ is used only during SSL (BYOL, DINO, MoCo v3), producing $p=g_{\emptyset }\left(z\right)$. A momentum (key) encoder mirrors the online encoder and is updated by exponential moving average (EMA) with momentum m=0.99, which is elaborated in Equation (2):

(2) $$\theta_{t+1}^{-}=m\theta_{t}^{-}+\left(1-m\right)\theta_{t}$$

In Equation (2) $\theta_{t}$ denotes the online network parameters at iteration t, while $\theta_{t}^{-}$ represents the parameters of the target network, updated using an exponential moving average (EMA):

On the other hand, SSL training minimizes a contrastive/predictive objective over two stochastic views T_1_ and T_2_ of the same patch, which is represented in Equation (3),

(3) $$L_{SSL}={Ε}_{\left({Χ}_{1},{Τ}_{1},{Τ}_{2}\right)}\left[l\left(g_{\phi }\left(f_{0}\left({Τ}_{1}\left(x\right)\right)\right),sg\left(g_{\phi^{-}}\left(f_{0}-\left({Τ}_{2}\left(x\right)\right)\right)\right)\right)\right]$$

where $l\left(g_{\emptyset }\left(f_{0}\left(T_{1}\left(x\right)\right)\right),sg\left(g_{\emptyset^{-}}\left(f_{0}-\left(T_{2}\left(x\right)\right)\right)\right)\right)$ is the method-specific loss (e.g., cosine/predictive for BYOL, InfoNCE for MoCo v3, centering/sharpening for DINO) and $sg\left(g_{\emptyset^{-}}\left(f_{0}-\left(T_{2}\left(x\right)\right)\right)\right)$ denotes stop-gradient as appropriate. We split the dataset into 80%training and 20%validation, retaining the natural per-class distribution (see Table 2). After SSL, encoder weights *θ* are transferred downstream.

For semantic segmentation, the SSL-pretrained backbone initializes DeepLabV3, which is fine-tuned on the same 64 × 64 labeled patches. Let *H* × *W* be the spatial size and ${∁}_{cls}$ the number of classes with logits $u_{i,c}$, and one hot labels $y_{i,c}$ at pixel 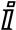. We minimize the loss in Equation (4):

(4) $$L_{seg}=-\sum_{i=1}^{H\;\times \;W}\sum_{c=1}^{C_{cls}}y_{i,c}log\left(softmax_{\left(u_{i,c}\right)}\right)$$

Final predictions on the Alpine Wetland imagery are generated using a sliding window over the full-resolution mosaics to ensure complete coverage.

To make the distinction explicit, we define a task factor represented in Equation (5):

(5) $$f\left(x\right)=\left\{\begin{array}{c} z=f_{0}\;\;\;\;\;\;\;\;\;\;\;\;\;\;\;\;\;\;\;\;\;\;\;\;\;\;\;\;\;\;\;\;\;\;\;\;\;\;\;\;\;\;\;\;\left(SSL\;Pretrainingrepresentation\;learning\right)\\ y=argmax\;\backslash softmax{\left(h_{0}\left(f_{0}\left(x\right)\right)\right)}_{c}\;\;\;\left(DeeplabV3:Pixel-wise\;labelling\right) \end{array}\right.$$

where $h_{\emptyset }$ is the segmentation head and all other hyperparameters follow the defaults of the respective SSL methods.

### 2.2. Comparing with Other SSL Methods

**Figure 1:**
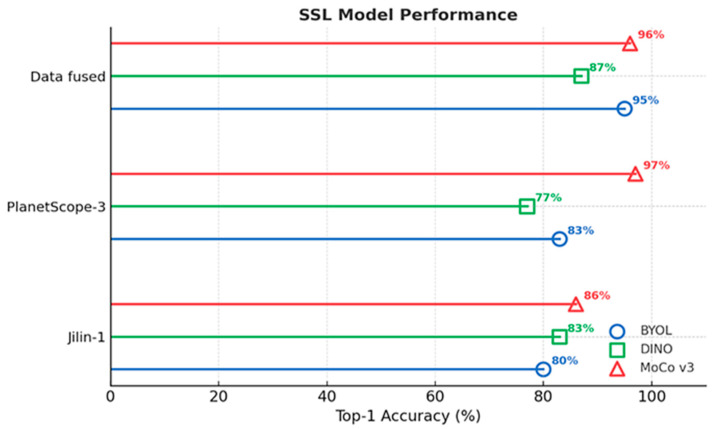
SSL model performance on Jilin-1, PlanetScope-3, and fused datasets using BYOL, DINO, and MoCo v3. MoCo v3 leads on single-sensor datasets, while BYOL performs best on the fused dataset.

On the Jilin-1 dataset, MoCo v3 achieved the highest accuracy of 86%TOP 1 OA, followed by DINO at 83% TOP 1 OA and BYOL at 80% TOP 1 OA. On the PlanetScope-3 dataset, MoCo v3 reported the best result with 97%TOP 1 OA, while BYOL and DINO reached 83%TOP 1 OA and 77% TOP 1 OA, respectively. For the fused dataset, BYOL achieved 95% TOP 1 OA, slightly lower than MoCo v3 at 96%, while DINO obtained 87%, as shown in Figure 1.

These results indicate that performance varied across methods and datasets. MoCo v3 consistently produced the highest scores on the single-sensor datasets (Jilin-1 and PlanetScope-3), while BYOL achieved the top result on the fused dataset.

### 2.3. Fine Tune SSL by DeeplabV3

**Figure 2:**
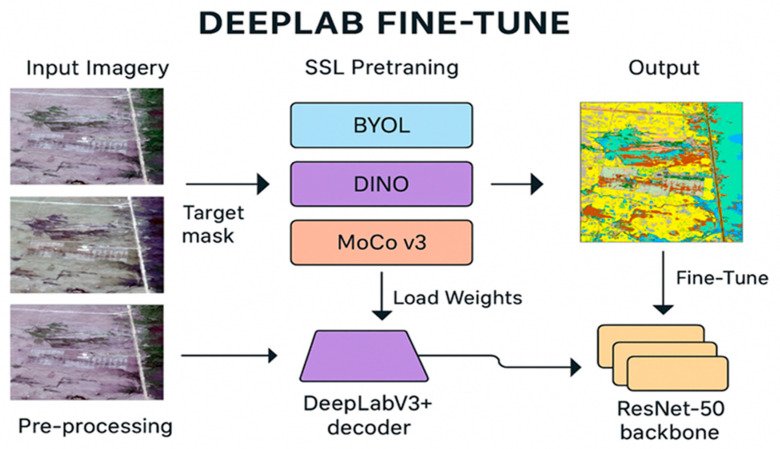
Workflow of the DeepLabV3+ fine-tuning framework. Multi-source satellite imagery is preprocessed and pretrained with BYOL, DINO, or MoCo v3, then transferred into a ResNet-50 backbone with a DeepLabV3+ decoder for segmentation map generation.

To achieve high-precision wetland vegetation mapping, we developed a fine-tuning pipeline built on DeepLabV3+ with a ResNet-50 backbone which is graphically shown in Figure 2. The workflow begins with multi-sensor satellite imagery, which is preprocessed and segmented into patches before being used for self-supervised pretraining with BYOL, DINO, or MoCo v3. These pretrained encoders provide robust feature representations, which are then transferred into DeepLabV3+ for supervised fine-tuning using annotated vegetation masks. The final output is a detailed segmentation map, where vegetation classes are clearly delineated and non-informative background pixels (class 0) are excluded from metrics. This approach demonstrates the benefits of SSL in boosting downstream segmentation performance in complex wetland environments.

**Figure 3:**
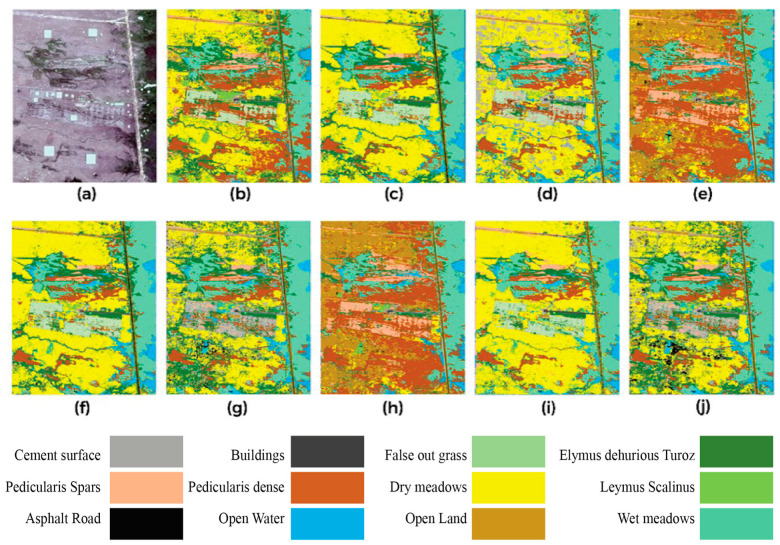
Semantic segmentation results of DeepLabV3+ fine-tuned with self-supervised pretrained backbones. (**a**) GT data, (**b**) Jilin-1 BYOL result, (**c**) Planet Scope 3 BYOL result, and (**d**) data fusion BYOL result; (**e**) Jilin-1 MoCo v3 result, (**f**) Planet Scope 3 MoCo v3 result, and (**g**) data fusion MoCo v3 result; (**h**) Jilin-1 DINO result, (**i**) Planet Scope 3 DINO result, and (**j**) Data fusion DINO result. The segmentation maps highlight diverse land cover types such as cement surfaces, buildings, pediculariids (sparse and dense), asphalt roads, open water, open land, meadows, and grassland types, with fused data often providing clearer and more accurate class boundaries than single-source imagery.

Moreover, the refined DeepLabV3 classification outcomes were obtained using different SSL approaches and datasets from the Bayinbuluke Alpine Wetland. The original Jilin-1 satellite, PlanetScope-3, and data fusion image are illustrated as follows: (a) Jilin-1 (BYOL), (b) Planet Scope 3 (BYOL), or (c) data fusion (BYOL); (d) Jilin-1 (MOCOv3), (e) Planet Scope 3 (MOCOv3), or (f) data fusion (MOCOv3); (g) Jilin-1 DINO, (h) Planet Scope 3 (DINO), or (i) data fusion (DINO). In the same way, the segmentation maps of MoCo v3 over Jilin-1, PlanetScope-3, and the fused dataset are provided in panel (g) to (i) in Figure 3.

Overall, the fused source delivers highly textured spatial information and enhances discrimination between vegetation classes—especially when distinguishing grassland associations (*Elymus dahuricus* and *Pedicularis* spp.) and differentiating meadow types. Quantitatively, BYOL provides the strongest segmentation quality overall, achieving the highest overall accuracy (O/A) on all three datasets and the best mIoU on Jilin-1 and the fused set, indicating sharper fine-scale boundaries. On PlanetScope-3, DINO attains the highest mIoU (65%), consistent with smoother, well-delineated boundaries on that sensor, although its O/A is lower than BYOL. MoCo v3 is competitive but slightly behind across sensors; its lower mIoU—particularly on PlanetScope-3—suggests under-segmentation of smaller classes when DeepLabV3 is trained on single-sensor inputs and accuracies metrices. The comparison indicates that DF improves class separability for most methods, with the BYOL-initialized model preserving the best balance between accuracy and boundary quality.

On the Jilin-1 dataset, BYOL achieved the highest performance with 93% overall accuracy and 64% mIoU, while DINO reached 76% overall accuracy and 56% mIoU, and MoCo v3 obtained 72% overall accuracy and 58% mIoU. For PlanetScope-3, BYOL again obtained the best overall accuracy (92%), whereas DINO produced the highest mIoU (65%); MoCo v3 followed with 86% overall accuracy and 61% mIoU. On the fused dataset, BYOL delivered the top scorers with 88% overall accuracy and 64% mIoU, closely followed by MoCo v3 at 87% and 63%, while DINO achieved the same overall accuracy (87%) but a lower mIoU of 57%, as shown in Table 1.

**Figure 4:**
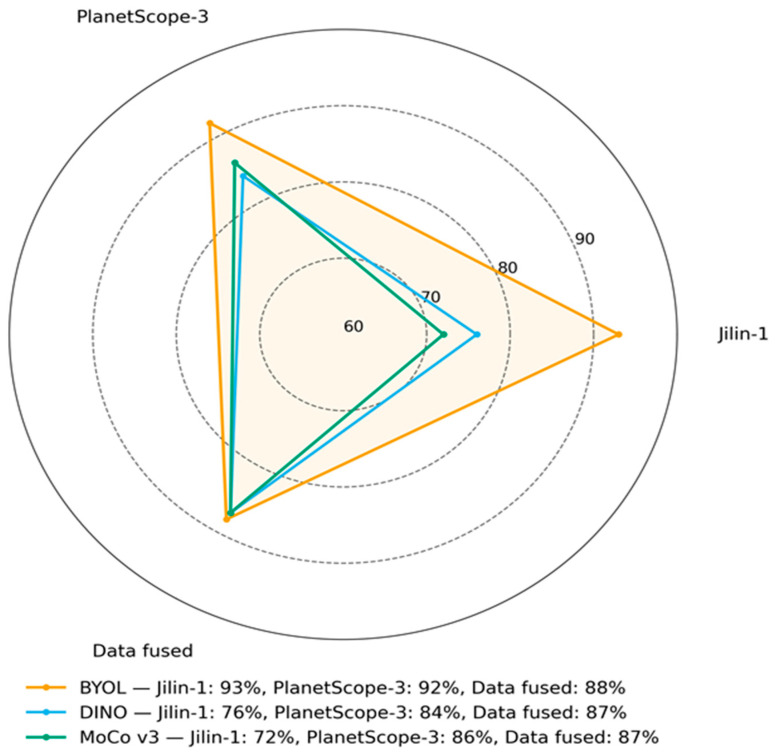
Radar chart of overall accuracy for BYOL, DINO, and MoCo v3 on Jilin-1, PlanetScope-3, and fused datasets.

However, radar chart (Figure 4) represents the overall accuracy between BYOL, DINO, and MoCo v3 on Jilin-1, PlanetScope-3, and fused datasets. The visualization allows us to see the performance differences according to various datasets and methods. The fused dataset gave the best result on the BYOL model (95%), and MoCo v3 yielded the best results on the single-sensor datasets (86% on Jilin-1 and 97% on PlanetScope-3). This indicates that SSL performance is dataset-specific, and MoCo v3 is better modeled on single-source datasets, while BYOL is good at multiple-sensor fusion.

## 3. Discussion

The results demonstrate that the performance of SSL methods is strongly influenced by both the input dataset and the downstream task. MoCo v3 consistently achieved the highest accuracies on the single-sensor datasets, particularly with PlanetScope-3 imagery, where its contrastive learning framework benefited from high temporal frequency and moderate spatial resolution. This finding is consistent with earlier research showing that contrastive SSL systems are especially effective when the spectral dimensionality of medium-resolution satellite images is limited but temporal variability is large [19,35]. This indicates that negative sample-based approaches are well suited for scenarios with limited spectral dimensionality but strong temporal variability. In contrastive SSL, a negative sample-based method is used whereby the model is trained to bring augmented views of the same image (positives) closer together in the feature space while pushing apart the representations of different images (negatives). This mechanism prompts the encoder to learn highly discriminative features that generalize well across diverse temporal measurements. By contrast, non-contrastive SSL designs such as BYOL rely on asymmetric network architectures and momentum updates rather than explicit negative pairs. The strong results of MoCo v3 on PlanetScope-3 therefore underline the advantage of negative sample-based contrastive learning in cases where spectral richness is low but temporal dynamics are high.

In contrast, BYOL had better performance on the fused dataset, demonstrating that predictive self-supervised learning without negative pairs works best in the high-dimension spectral–spatial setting. Similar trends have been observed in hyperspectral and multisensor applications, where redundancy among channels reduces the benefits of contrastive learning [17]. In contrast to methods that risk collapsing features when features are redundant, BYOL is actually able to make good use of complementary spectral and spatial information, without any requirement to define a negative set. This property favors BYOL being precisely appropriate in data fusion applications in the context of both redundancy and complementary information among sensors.

DINO exhibited mixed performance. While it underperformed on Jilin-1 and showed instability across datasets, it achieved the highest mIoU on PlanetScope-3, suggesting that self-distillation can help preserve boundary quality under certain imaging conditions. Similar improvements in boundary delineation have been reported in remote sensing segmentation tasks [36,37]. Although the overall sensitivity to dataset characteristics reduces its robustness compared with BYOL and MoCo v3.

The segmentation results further highlight the benefits of SSL pretraining. Across all methods, SSL-initialized DeepLabV3 models produced sharper boundaries and clearer class separation than purely supervised training with limited ground-truth, consistent with findings in ecological and agricultural remote sensing [38,39]. Data fusion consistently improved the identification of minority and fragmented classes, such as Elymus- and Pedicularis-dominated patches, wet meadows, and invasive species. These improvements are ecologically significant, as the ability to delineate rare vegetation types and invasive species is critical for biodiversity monitoring and conservation management. Prior research has pointed out the difficulty of targeting invasive species because they share spectral characteristics with other vegetation present [8,10,40]. The fact that fusion-SSL methods did work in this case illustrates the fact that it is possible to overcome this difficulty.

This research has several limitations although the results are promising. The use of only one point in time (June 2024) represents the vegetation during its peak season without accounting for the dynamics of phenology throughout the year. These limitations could prevent the identification of species that have a characteristic seasonal pattern, such as invasive plants that grow at varying stages. It could also be more relevant to include multi-temporal datasets that have been used to perform good vegetation classification and invasion species detection [41,42], to bring a broader perspective of the wetland dynamics. The ground-truth volume of data is also a limitation as it is relatively small. Although through the use of SSL, there is less reliance on annotated samples, the small number of field observations may influence the overall validity of the framework in its application to other wetlands.

These difficulties should be addressed in future studies by increasing field sampling and using semi-supervised or active learning to tradeoff between the cost of annotation and the quality of validation. To further enhance robustness of the sensors in heterogeneous wetlands, integration of complementary sensors including synthetic aperture radar (SAR) and LiDAR, which record structural and backscatter data, can be employed. The transferability of the framework across various ecological and imaging conditions would also be possible by applying the framework to different wetland systems. The application of transfer learning, UAV validation, and closer integration with biodiversity monitoring programs would scale the approach and make it more applicable for long-term conservation and ecological management.

## 4. Material and Methods

### 4.1. Study Area

**Figure 5:**
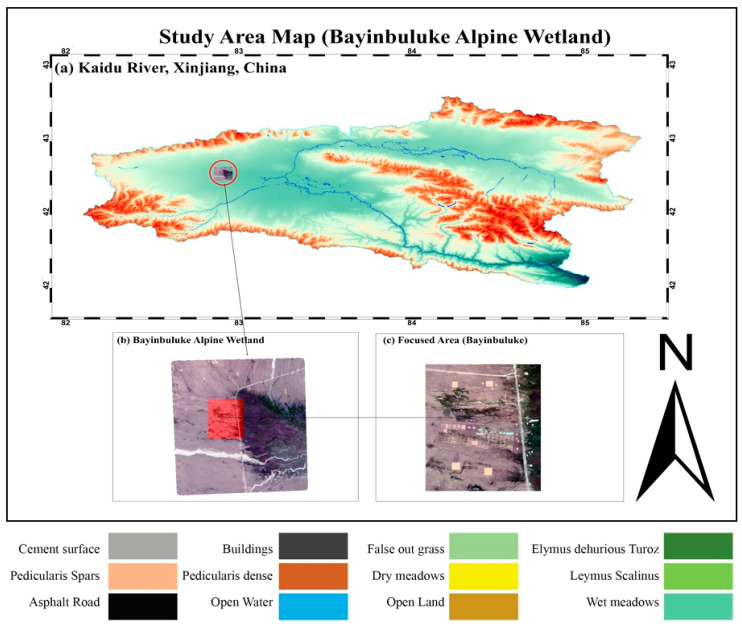
Location of the Bayinbuluke Alpine Wetland in Xinjiang, China, with panels showing (**a**) Kaidu River, Xinjiang, China, (**b**) Bayinbuluke Wetland, and (**c**) the focused study area. The legend indicates major land cover and vegetation classes used in this study.

The study area is the Bayinbuluke Wetland, located in the Kaidu River Basin in the central Tianshan Mountains of Xinjiang Uyghur Autonomous Region, northwest China, as shown in a Figure 5. The Bayinbuluke Wetland (b) subregion used for classification and analysis is identified in Figure 5c as the focused area of Bayinbuluke. Moreover, it extends between 42.78°to 43.23° latitude and 83.68° to 84.27° longitude with an average elevation of 2400 m, characteristics of a high-altitude alpine basin. The region has a cold temperate continental climate, which is manifested by long, cold winters and short, warm summers. The average monthly temperatures are as low as −20 °C in January to as high as 15 °C in July, and the mean annual precipitation is 270 mm, mostly from May to September. Potential evapotranspiration exceeds more than 1000 mm annually, and glacial meltwater together with seasonal rainfall, sustains wetlands inundation and soil saturation, peaking in spring and early summer.

Bayinbuluke Wetland is one of the largest alpine wetlands in China and forms part of the Bayanbulak Grassland National Nature Reserve. It supports a mosaic of ecosystems including alpine meadows, marshes and rivers, and shallow lakes that provide critical breeding habitats for migrating birds, notably the endangered Black-necked Crane (*Grus nigricollis*). Vegetation includes cold-adapted grasses, herbaceous plants, and lowland meadows tolerant of flooding. The dominant land cover consists of alpine grasslands (~65%), marsh wetlands (~20%), and open water (~5%), along with limited rocky and gravel surfaces.

For this study, a subregion of Bayinbuluke Wetland (Figure 5c was selected for land cover classification using remote sensing and machine learning. Twelve vegetation and land cover classes were defined, including natural meadows, open water, developed surfaces, and herbaceous vegetation types, some of which represent known invasive species. This scheme enables accurate delineation of habitat composition and structure, forming the basis for subsequent segmentation modeling and ecological analysis.

### 4.2. Data and Processing

#### 4.2.1. Satellite Imagery

We used two high-resolution satellite datasets PlanetScope-3 (3 m) and Jilin-1 (4 m), as the main inputs for wetland classification. The PlanetScope-3 constellation captures imagery across eight spectral bands, including coastal blues, blue, green, yellow, red, red edge, and two near-infrared (NIR), while the Jilin-1 satellite provides four bands including blue, green, red, and near-infrared (NIR). The overall data was obtained in June 2024, which was the peak of the growing season in Bayinbuluke when vegetation differences are the most significant. The data on satellite imagery, topography, proximity, and reference data employed in this study are reported in detail in Table 2.

Moreover, DF was performed to enhance the classification accuracy as well as the spectral richness by combining the two satellite sources to provide one dataset having 11 spectral bands to better classify the spectral information and greater spectral detail. Geometric correction, co-registration, and resampling to a common spatial resolution were performed to preprocess all imagery prior to fusion. This kind of combination of complementary spectral signatures enabled a more precise sorting of wetland vegetation and land cover categories. Moreover, overview of datasets and supporting information were used for wetland vegetation classification, including satellite imagery, topography, proximity, and reference data.

**Figure 6:**
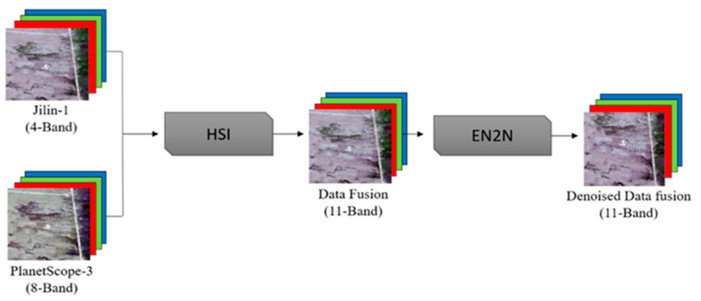
Data fusion workflow combining Jilin-1 (4-band) and PlanetScope-3 (8-band) imagery. The images are first fused using the HSI method to generate an 11-band dataset, followed by EN2N-based denoising to produce the final fused data.

The data fusion output was then denoised through a convenient self-supervised SAR denoising model using the EN2N model, a self-supervised SAR denoising framework, which aims to represent a convolutional neural network (CNN) adapted during the current study to the domain of optical images preprocessing [43]. The denoising procedure maintained the spatial integrity of an image by minimizing the noise component, thus enhancing the quality of the dataset to which the classification would be applied, as described in Figure 6.

#### 4.2.2. Topographic Information

Bayinbuluke Wetland in Xinjiang, located in the central Tianshan mountain of China, is one of the largest wetlands of the alpine regions in the country and is a component of the Bayanbulak Grassland National Nature Reserve. It lies at an average of about 2400 m above sea level with alpine meadows, marshes, shallow lakes, and riverine systems which offer important breeding areas to migratory birds such as the endangered Black-necked Crane (Grus nigricollis). The most common land cover types are alpine grasslands (more than 65 percent), marsh wetlands (more than 20 percent), open water (more than 5 percent), and small rocky and gravelly surfaces. The vegetation community comprises cold-adapted grasses, herbaceous plants, and meadow types partially adapted to low temperatures and seasonal flooding.

The basin topography is characterized by flat to gently undulating alluvial plains surrounded by steep mountains exceeding 4000 m. These slopes generate glacial and snowmelt runoff, which feeds into the Kaidu River and its tributaries, maintaining soil saturation and extensive marsh formation. Water retention is especially pronounced in the low-gradient central wetland zones, where slopes are generally <3°. Over centuries, meltwater deposition from Tianshan streams has shaped the meadows, marshes, and shallow river systems observed today.

The measurements of topographic variables, e.g., elevation, slope, and hydrological connectivity, were determined based on the digital elevation models (DEMs), i.e., Shuttle Radar Topography Mission (SRTM) and Advanced Spaceborne Thermal Emission and Reflection Radiometer Global DEM (ASTER GDEM). These datasets were used to classify the sub-regional land cover and for further ecological analysis.

#### 4.2.3. Proximity Information

The Bayinbuluke Wetland is located along the headwaters of the Kaidu River, which is one of the major tributaries draining glacial meltwaters from surrounding highlands. Proximity to the Kaidu River and its distributaries is critical for seasonal hydrological processes in the wetland, particularly in periods of spring and early snowmelt of summer. The region is characterized by its remoteness and limited human disturbance, with few settlements scattered along the outer edge of the reserve. The area is lowly inhabited, remote, and not heavily disturbed by man, with occasional settlements found on the perimeter of the reserve. It is near Hejing County, about 200 km to the northwest, and about 500 km to Urumqi, the capital of Xinjiang. The ecological integrity of this area is maintained by the presence of the wetland with proximity to snow-capped mountains, and its distance from areas of intensive agriculture or urban expansion.

In this research, the proximity analysis was carried out with the help of the GPS coordinates gathered during field work in June 2024, coupled with the geographical characteristics of the area being taken from OpenStreetMap (OSM) and high-resolution imagery. These sources provide reliable spatial points of reference to map the boundaries of the wetlands and the surrounding streams, roads, and features of the landscape. Major hydrological references were made based on the Kaidu River that happens to wind through the basin. All the proximity layers were processed in GIS environment for analysis. Calculation of field GPS data and OSM data enabled the fine definition of wetland extents, buffer areas, and spatial relationships between vegetation zone and drivers of the environment, i.e., water bodies and elevation gradients.

#### 4.2.4. Reference Data

This study used a combination of field-collected Global Positioning System (GPS) coordinates and vector data obtained via OpenStreetMap (OSM) as the reference data. The GPS points (the number of which was obtained across representative land cover types in June 2024) were collected by field teams and then cross-validated with high-resolution imagery and OSM-derived features in order to ensure spatial accuracy and contextual consistency in our data. Using this geospatial information, polygon shapefiles have been created manually by digitizing all 13 land cover and vegetation classes in the study area, including natural and artificial surfaces, as shown in Table 3.

These class polygons serve as the foundation for supervised learning utilizing the labeled regions, from which we extracted 64 × 64-pixel image patches from the fused 11-band data (PlanetScope-3 and Jilin-1) images so that each patch occupies the same spatial location as the label that it corresponds to. The procedure facilitated the creation of an organized dataset intended for subsequent picture classification and semantic segmentation via deep learning, while maintaining label integrity and minimizing overlap among classes at the boundaries between patches.

In order to take advantage of both sensors, the two datasets were co-registered and fused with each other spatially. The bands of PlanetScope-3 and Jilin-1 were merged together, and they formed a fused image with 11 distinctive spectral bands. The spectral richness and spatial details of the fused dataset help attain a high accuracy in classification of wetland vegetation.

#### 4.2.5. Methodology Workflow

This research workflow includes three stages that are connected with one another, namely: data preparation, feature engineering, and modeling. The first step involved obtaining multi-source satellite imagery from PlanetScope-3 and Jilin-1, as well as ground-truth data in the form of shapefiles, GPS surveys, and raw field observations. The two satellite datasets were merged into an 11-band fused image by using a DF process, followed by preprocessing, co-registration, and cropping of the fused image along with their respective labels into 64 × 64 patches.

**Figure 7:**
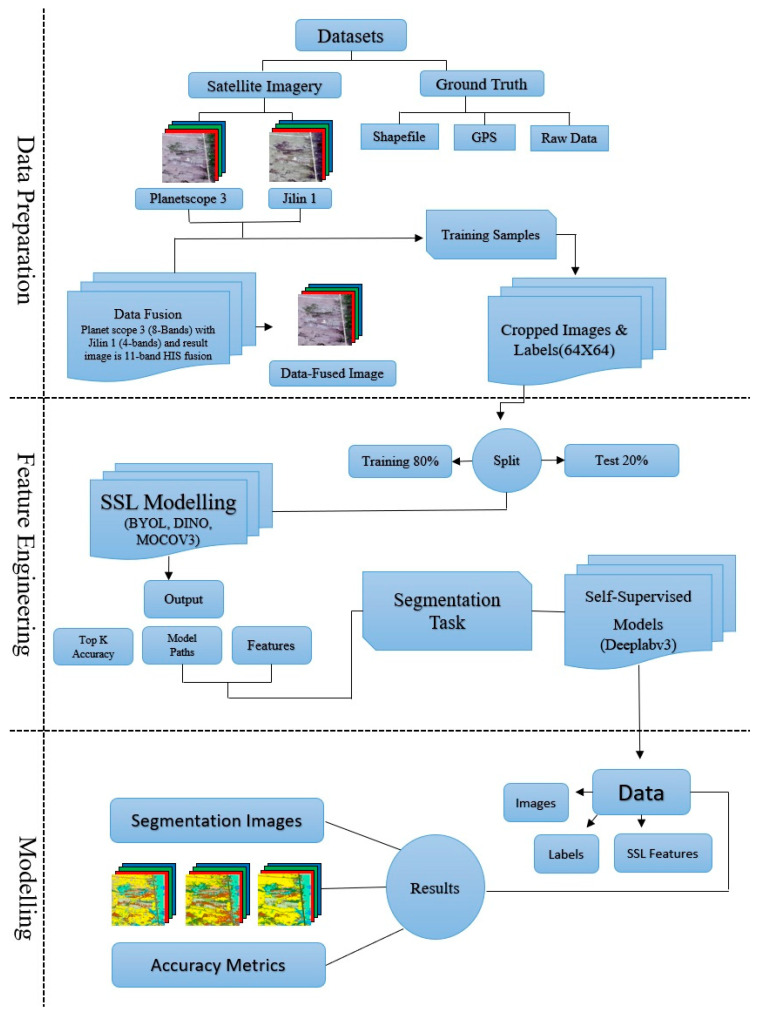
Workflow of data preparation, SSL feature extraction (BYOL, DINO, MoCoV3), and segmentation with DeepLabv3 for accuracy evaluation.

To train the dataset, 80 percent of the data were used as a training sample and 20 percent were used as a validation sample to ensure that a robust model is developed. The self-supervised learning (SSL) models BYOL, DINO, and MoCo v3 were trained on fused datasets and single-source datasets over 100 epochs with a ResNet-50 backbone during the feature engineering stage. Their representations were then gauged using a linear probe that had been trained in 100 epochs, and Top-k accuracies were collected to test the quality of features represented. The general workflow is shown in Figure 7.

During the last modeling phase, the trained SSL backbones were transferred to a DeepLabV3 segmentation model. The model was adjusted with 100 epochs on the labeled 64 × 64 patches, the ignore-0 index was used during the training to deal with the background pixels. The final resultant segmentation maps of the Bayinbuluke Wetland using the DeepLabV3-pretrained with the SSS were high-resolution. The overall accuracy (O/A) and mean Intersection over Union (mIoU) were used as metrics to determine model performance when comparing various methods of applying SSL and data fusion.

#### 4.2.6. Computational Environment

All experiments were conducted on a workstation equipped with an Intel processor-based workstation with 64 GB RAM and NVIDIA GeForce RTX 4060, 8 GB VRAM, and Ubuntu 22.04 LTS. We conducted all experiments in PyCharm (2024.1), with the environment managed in Anaconda using Python 3.12.11 (conda-forge build, MSC v.1943, 64-bit),PyTorch 2.4.1 + cu124 was used to develop and evaluate the models with CUDA 12.4 turned on. The graphics card was identified as NVIDIA GeForce RTX 4060. The additional libraries were Torchvision 0.19.1+cu124, NumPy 1.26.4, Matplotlib 3.10.6, Lightly 1.5.20, and scikit-learn 1.7.2, Rasterio 1.4.3, and ArcGIS Pro were also used to complete geospatial data processing and visualization as summarized in Table 4.

In self-supervised pretraining (BYOL, DINO, MoCo v3), models were trained for 100 epochs with a batch size of 64, a learning rate of 1 × 10^−4^, and a weight decay of 1 × 10^−4^ were used. These conditions were typical of use in the literature and had been adjusted by preliminary experiments in order to stabilize training. Average pretraining time was approximately 1 h 45 min (±5 min) and linear evaluation took about 45 min (±5 min) per model.

For downstream semantic segmentation, the SSL-pretrained backbones were transferred to a DeepLabV3 model trained for 100 epochs, using the ignore-0 index to exclude background pixels. Fine-tuning required around 2 h (±10 min) per model. The conditioning of 64 × 64 patches was nearly real time (less than 1 s), enabling large-scale mapping of the Bayinbuluke Wetland.

## 5. Conclusions

This study demonstrated that integrating SSL with supervised segmentation and data fusion can deliver high-resolution wetland vegetation mapping with a significant improvement in accuracy. The tested techniques performed best on single-sensor and fused data, respectively, with MoCo v3 performing best on single-sensor data, whereas BYOL performed best on fused data, underscoring the need to tailor SSL approaches to data properties. DINO displayed promise with regard to establishing boundaries but lacked robustness across the datasets. The pretraining consistently improved the quality of segmentation, especially in minority and fragmented classes, whereas data fusion improved recognition of rare and invasive vegetation types. The findings mean that SSL limits the need to rely on large amounts of field labeled data and offers a feasible platform for complicated wetland environments. However, they have limitations regarding time coverage as well as the lack of ground-truth. The combination of SSL with the multi-sensor fusion has great potential as an avenue toward generating meaningful and operationally useful information on wetland vegetation.

## Figures and Tables

**Table 1:** Overall accuracy (O/A) and mean Intersection over Union (MIoU) achieved by BYOL, MoCoV3, and DINO on Jilin-1, PlanetScope-3, and fused datasets, highlighting the superior performance of MoCoV3 on fused imagery.

Datasets	BYOL (O/A)	BYOL (mIoU)	MoCoV3 (O/A)	MoCoV3 (mIoU)	DINO (O/A)	DINO (mIoU)
Jilin-1	93%	64%	72%	58%	76%	56%
PlanetScope-3	92%	63%	86%	61%	84%	65%
Data fusion	88%	64%	87%	63%	87%	57%

**Table 2:** Overview of datasets and supporting information used for wetland vegetation classification, including satellite imagery, topography, proximity, and reference data.

Data	Description	Application Data
Satellite Imagery	High-resolution optical imagery acquired in June 2024 from PlanetScop-3 and Jilin-1 for wetland classification.	PlanetScope-3 (9-Band Image), Jilin-1 (4-Band Image) or data-fused image (PlanetScope-3 (8-band) + Jilin-1 (4-Band) = 11-Band-Fused Image).
Topographic Information	High-altitude alpine basin (~2400 m elevation) with flat valley floors surrounded by the Tianshan Mountains; predominantly marshes and meadows.	Alpine basin (~2400 m), whose valley floors are flat and is surrounded by Tianshan mountains; water accumulation provides the setting of marshes and alpine meadows.
Proximity Information	Proximity data from field-collected GPS data and OpenStreetMap (OSM)—includes wetland boundary information, water bodies and nearby landscape features.	Applied to study spatial relationships between wetlands, water bodies, and the surrounding land features; supports habitat mapping and hydrological modeling.
Reference Data	Reference data were generated using field-collected GPS points and OpenStreetMap (OSM) vectors. Polygons for 13 land cover and vegetation classes were manually digitized and cross-verified using high-resolution imagery. These served as ground-truth for supervised learning.	Supports training and validation of deep learning models for land cover classification and segmentation; ensures label accuracy and spatial alignment across image patches.

**Table 3:** Distribution of ground-truth samples across 12 land cover and vegetation classes, totaling 8892 labeled instances.

ID	Class Names	Image Samples
1	Cement surface	526
2	Buildings	668
3	False out grass	985
4	Elymus dehurious Turoz	531
5	Pedicularis sparse	568
6	Pedicularis dense	1026
7	Dry meadows	2522
8	Leymus Scalinus	200
9	Asphalt road	567
10	Open water	269
11	Open land	334
12	Wet meadows	1383
Total		8892

**Table 4:** Computational environment and training settings.

Category	Specification
Hardware	Intel CPU, 64 GB RAM, NVIDIA GeForce RTX 4060 (8 GB VRAM)
Operating System	Ubuntu 22.04 LTS
Python	3.12.11 (Conda-Forge, MSC v.1943 64-bit)
PyTorch	2.4.1 + cu124
CUDA	12.4
GPU Device	NVIDIA GeForce RTX 4060
Libraries	Torchvision 0.19.1+cu124, NumPy 1.26.4, Matplotlib 3.10.6, Lightly 1.5.20, scikit-learn 1.7.2, Rasterio 1.4.3, etc.
Software	ArcGIS Pro
SSL Training	100 epochs, batch size = 64, LR = 1 × 10^−4^, weight decay = 1 × 10^−4^
Training Time	Pretraining: ~1 h 45 m (±5 m), Linear evaluation: ~45 m (±5 m), Fine-tuning: ~2 h (±10 m)
Path Inference	64 × 64 patches, <1 s (real time)

## Data Availability

Data are available from the corresponding author upon reasonable request.
